# mRNA-Seq and microarray development for the Grooved carpet shell clam, *Ruditapes decussatus*: a functional approach to unravel host -parasite interaction

**DOI:** 10.1186/1471-2164-14-741

**Published:** 2013-10-29

**Authors:** Ricardo B Leite, Massimo Milan, Alessandro Coppe, Stefania Bortoluzzi, António dos Anjos, Richard Reinhardt, Carlos Saavedra, Tomaso Patarnello, M Leonor Cancela, Luca Bargelloni

**Affiliations:** 1 CCMAR- Center of Marine Sciences/University of Algarve, Campus de Gambelas, 8005-139 Faro, Portugal; 2 Department of Comparative Biomedicine and Food Science, University of Padova, I-35020 Legnaro, Italy; 3 Biology Department, University of Padova, Via G. Colombo 3, I-35131 Padova, Italy; 4 ISMAT- Instituto Manuel Teixeira Gomes, Avenida Miguel Bombarda nº 15, 8500-508 Portimão, Portugal; 5 Max Planck Institute for Molecular Genetics, Ihnestraße 63-73, 14195 Berlin, Germany; 6 Instituto de Acuicultura de Torre la Sal (IATS), Consejo Superior de Investigaciones Cientificas (CSIC), 12595 Ribera de Cabanes, Castellón, Spain; 7 DCBM Department of Biomedical Sciences and Medicine, University of Algarve, 8005-139 Faro, Portugal; 8 Current address: Instituto Gulbenkian de Ciência, Rua da Quinta Grande, 6, 2780-156 Oeiras, Portugal

## Abstract

**Background:**

The Grooved Carpet shell clam *Ruditapes decussatus* is the autochthonous European clam and the most appreciated from a gastronomic and economic point of view. The production is in decline due to several factors such as Perkinsiosis and habitat invasion and competition by the introduced exotic species, the manila clam *Ruditapes philippinarum*. After we sequenced *R. decussatus* transcriptome we have designed an oligo microarray capable of contributing to provide some clues on molecular response of the clam to Perkinsiosis.

**Results:**

A database consisting of 41,119 unique transcripts was constructed, of which 12,479 (30.3%) were annotated by similarity. An oligo-DNA microarray platform was then designed and applied to profile gene expression in *R. decussatus* heavily infected by *Perkinsus olseni.* Functional annotation of differentially expressed genes between those two conditionswas performed by gene set enrichment analysis. As expected, microarrays unveil genes related with stress/infectious agents such as hydrolases, proteases and others. The extensive role of innate immune system was also analyzed and effect of parasitosis upon expression of important molecules such as lectins reviewed.

**Conclusions:**

This study represents a first attempt to characterize *Ruditapes decussatus* transcriptome, an important marine resource for the European aquaculture. The trancriptome sequencing and consequent annotation will increase the available tools and resources for this specie, introducing the possibility of high throughput experiments such as microarrays analysis. In this specific case microarray approach was used to unveil some important aspects of host-parasite interaction between the Carpet shell clam and *Perkinsus*, two non-model species, highlighting some genes associated with this interaction. Ample information was obtained to identify biological processes significantly enriched among differentially expressed genes in *Perkinsus* infected versus non-infected gills. An overview on the genes related with the immune system on *R. decussatus* transcriptome is also reported.

## Background

European clam aquaculture production is centered in three major species of clams: *Ruditapes philippinarum*, the manila clam, *Ruditapes decussatus*, the grooved carpet shell clam and *Venerupis pullastra*, the pullet carpet shell clam. According to FAO and Fishstat reports, most of the relevant increases of production have been concentrated in *R. philippinarum* and *R. decussatus,* both of which have been severely affected by perkinsosis during the last years. Historical records show that *R. decussatus* was one of the major aquaculture species in Europe, but due to overfishing, recruitment failures and some outbreaks of bacterial infection and parasitism, producers started to substitute this species for a closer but exotic clam from the same family, the manila clam *R. philippinarum*[1]. The introduction of this species, with a faster growing rate and believed to be more resistant to some diseases, originated a progressive replacement of the native clam and nowadays the production of grooved carpet shell clam is almost insignificant in most Mediterranean countries. However, its commercial, historical and gastronomic values are still high making the production of this clam an important niche to explore. Despite the fact that its culture decreased considerably in countries such as France, Italy, Portugal and Spain, there is some desire to increase *R. decussatus* production. Yet, because of the potentially high vulnerability of its production, management and control strategies and their implications when implemented are key factors for protecting this industry from the effects of diseases. One of the most persistent infections is caused by the parasite *Perkinsus sp.,* a facultative intracellular protist parasite belonging to the phylum Perkinsozoa. Interestingly, *Perkinsus* is also considered a model organism to understand adaptations to parasitism [2].

Parasites from *Perkinsus sp.* family are considered to be one of the most problematic agents being blamed for mass infections leading to dramatic reductions in culture beds of clams *Ruditapes descussatus* in Southern Portugal [3,4]. Some reports indicated mortalities up to 80% in Portugal, with more than 90% of the clams infected in a specific season/area. Also Spain and France reported high mortality rates reaching up to 100% in cultures of Manila clam in Spain [5]. Although clam Perkinsiosis was first identified in *R. decussatus*, the Manila clam can also be affected and *Perkinsus* was first detected in South Korea in 1993 [6] and later in China [7] and Japan [8]. The agent responsible for these mortalities, *Perkinsus olseni* is the same that caused similar episodes in Europe but it can also infected abalones, pearl oysters, oysters and other species of clams besides *Ruditapes* sp. Although the clam infection process is not entirely unveiled, it starts when the *Perkinsus* trophozoites, a free living stage, are uptake by the host, followed by their engulfment by bivalve hemocytes in which are capable of remaining viable, proliferate by successive bipartitioning in the connective tissue of all organs and disseminate throughout the entire organism [9], leading to host death in most cases. In the case of infection, lectins are the main mechanism responsible to trigger bivalve’s defenses by recognizing and preventing infection [10-13]. Other mechanisms are involved such as the generation of proteases inhibitors [14], lysosomal enzymes and ROS species and parasite encapsulation [15].

The routine use of high throughput sequencing and microarrays is becoming more frequent and it is revolutionizing the study of host-parasite interaction [16-19] revealing some key molecular interactions and modulation of host-species to parasite. The scientific community is paying more attention to marine organisms and in the last 3–4 years some important commercial species of fish and shellfish were sequenced and became the target of gene expression studies [20-26]. The importance of mollusks as biological filters and thus potential bio-monitors cannot be sub estimated and this tool can also be applied to infer some facts about how pollution and other antropogenic activities can influence clam transcriptome, increasing the range of future applications for the platform presented here. In the present study we aimed to infer how a parasite can influence host gene regulation by looking at host gene profile and expression and interpreting the basis of molecular determinants by pinpointing host gene clusters, processes and mechanisms of defense and co-existence with the parasite. In conclusion we present a set of new tools for the grooved carpet shell clam, comprising a transcriptome survey, web database integrating gene annotation and blast search and the introduction of an adaptable microarray platform for *R. decussatus*.

## Results and discussion

### Next-generation sequencing and hybrid contig assembly

Using Roche 454 FLX technology, two sets of libraries (MGE011: 122,471 reads; cDN18: 327,209 reads) consisting of a total of 449,680 reads were sequenced using normalized cDNA libraries constructed using either a mixture of adult tissues or containing gonadal tissue and entire larvae. The same libraries were used to obtain respectively 2,434 and 2,077 ESTs with traditional Sanger sequencing analysis. Using all data available, amounting to a total of 454,191 reads plus ESTs, a assembly was performed and grouped them into 41,119 contigs.

**Figure 1:**
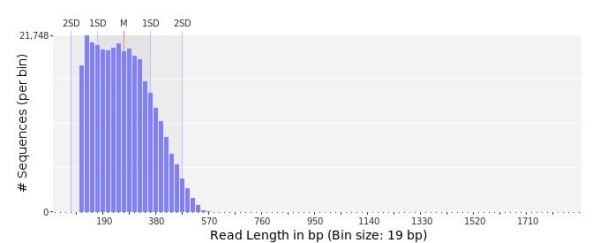
**Read length distribution of *R. decussatus *454 sequencing.** Bars indicate relative percentage of reads per real length in an interval of 19 bp, with an average read length of 257 bp.

**Figure 2:**
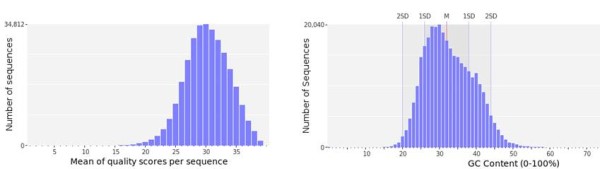
**Reads quality score graphic (left) and GC content in percentage (right).** Bars indicate relative percentage of reads per quality (left) and GC content (right).

The average read size from 454 sequencing was 257 bp (Figure 1 shows the distribution of sequence lengths) and quality level of the reads was assured by a distribution of the sequences with 96% of reads with Phred sequence quality >20 (Figure 2, left panel) while ESTs of *R. decussatus* have a mean length of 604 bp. The GC content of the reads was in average 33% ± 6,6% similar with the EST sequences of *R. decussatus* deposited in Genbank (34.78 ± 6.26%), with a maximum of 74% (Figure 2, right panel). All 454 reads have been deposited in GenBank (SRA) with the accession number [SRA058431].

### Transcriptome annotation and microarray quality assessment

To determine the putative identities of assembled contigs, Blastx and Blastn similarity searches on several protein and nucleotide sequence databases were performed. Of 41,119 unique sequences, 8,560 (21%) showed at least one significant match (e<10^-3^) in the NCBI non-redundant protein database (Additional file 1). In addition to the annotation with Blast2GO, Blast searches against UniProtKB/Swiss-Prot database, UniProtKB/TrEMBL database and 5 different species-specific data bases (Additional file 1) were implemented in order to further increase the number of putatively annotated *R. decussatus* contigs (see Methods for details)*.* This approach provided a significant match for additional 3,919 transcripts, which previously showed no correspondence with the NCBI non-redundant protein database, bringing the final number of clam entries associated with a known protein or transcript to 12,479 (30.3%). The percentage of annotated expressed sequences is very similar to that obtained for *R. philippinarum* (30%) by Milan and co-workers [24]. The highest number of significant similarity scores (9,364 hits, 22.8%) was obtained with *Crassostrea gigas* protein database, second best-matching species *Lottia gigantea* (8,828 hits, 21.4%) and third *Danio rerio* (7,170 hits, 17.4%) in accordance to what was previously observed for *R. philippinarum* annotation (24,1% and 18.5% annotated contigs with *L. gigantea* and *D. rerio* respectively).

Probe design took into consideration all annotated entries (12,479). Non annotated transcripts with sequence lengths ≥400 bp and average Phred sequence quality > 30 were also considered. A total of 21,900 target sequences were obtained and for each of them, two probes with opposite orientations (sense and antisense) were designed. A total of 43,758 out of 43,800 (99.9%) probes were successfully obtained, representing 21,887 *R. decussatus* transcripts. The percentage of annotated transcripts represented in the microarray was 57%. Annotated genes were categorized according to Gene Ontology (GO) Functions in the three root categories and also in terms of main families of genes using a previously defined GO classification (see Additional file 2). Probe sequences and other details on the microarray platform can be found in the GEO database under accession number GSE36276.

### Validation of microarray data by quantitative real-time PCR analysis of gene expression

**Figure 3:**
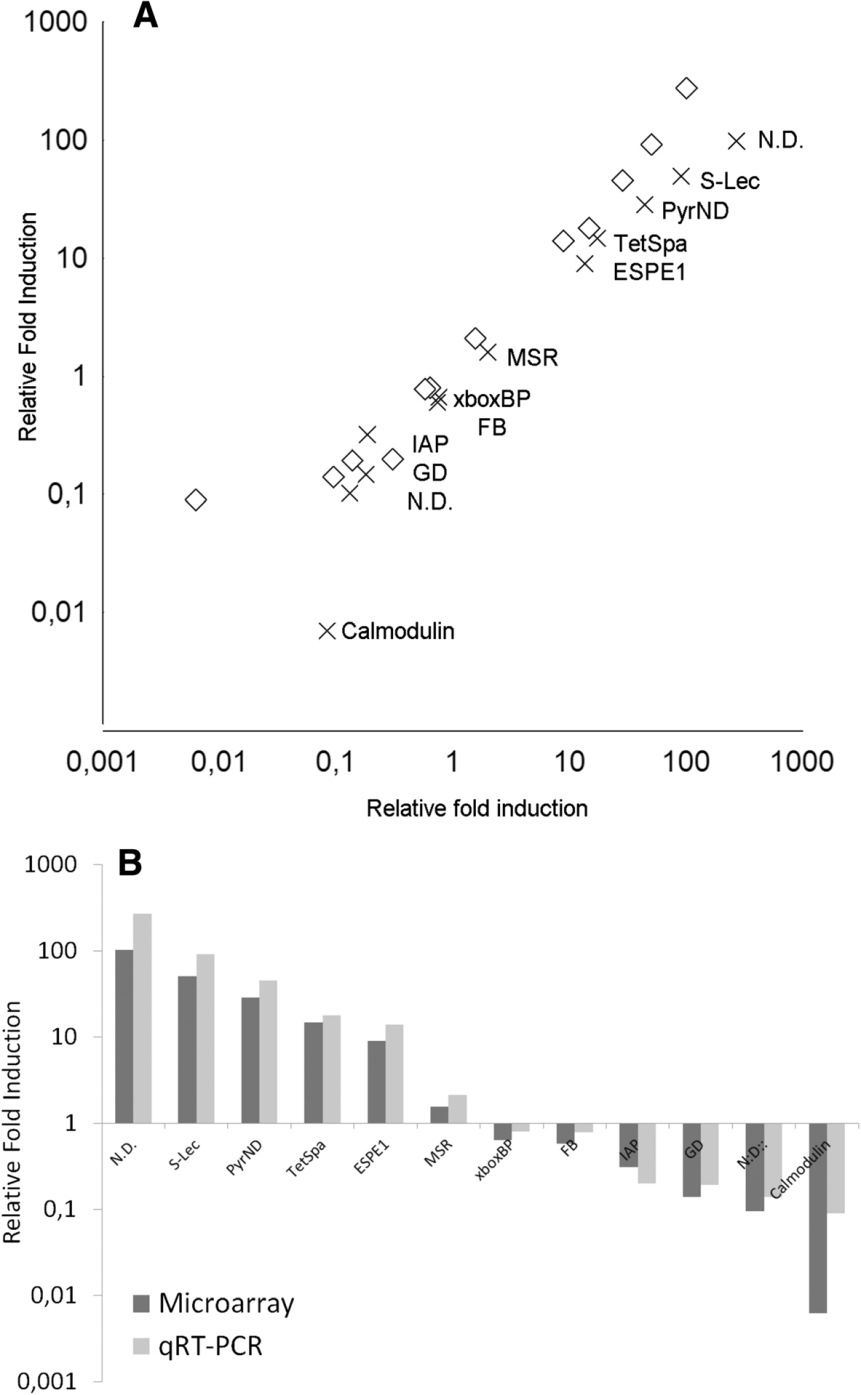
**Combined plot representation (log scale) of Pearson correlation matrixes of microarray probe fold change (crosses) and respective qPCR (diamonds) (A) and Relative Fold Induction of microarray probe fold change (dark grey bar) and respective qPCR (light grey bar) (B) upon presence or absence of infection in selected sequences/probes.** N. D. Stands for Sequence not determined, S-Lec for sialic acid-binding lectin; PyrND for pyridine nucleotide-disulphide oxidoreductase; TetSpafor tetraspanin, ESPE1 for Epididymal secretory protein E1: MSR for methionine sulfoxide reductase b3 isoform 2 isoform 1xboxBP for x-box binding protein 1; FB for fructose- -bisphosphatase; IAP for inhibitor of apoptosis protein; GD for glucose dehydrogenase and calmodulin.

Comparative analysis of microarray and qPCR expression data is presented in Figure 3. Pearson correlation indicated coefficients of 0.984 for 12 compared genes and a p <0.005 when comparing data for microarray probe and correspondent qPCR, corroborating the good reliability of the microarray platform. Fold change is always higher by qPCR than measured by microarray with one exception, a common situation with Agilent microarrays [27].

### Microsatellite content (SSR-EST)

The genome of Bivalves is known to harbor a large number of microsatellites [28], that can be useful for as markers for different kinds of studies such as population genetic structure, demography, selective breeding and quantitative trait loci studies in clam [29]. Nevertheless microsatellite marker development can be difficult to develop in mollusk for many reasons, some of them still unknown [30]. EST derived microsatellites have some advantages such as being more conserved across species and be more adequate for selective pressure studies for example [31]. Previous studies have characterized some EST-SSR in clams using 454 [26,32] but none used *R. decussatus* as a model.

We used two similar approaches to determine if microsatellites are transcribed into *R. decussatus* RNA, one using Misa script [33] and Msatcommander [34]. Table 1 reflects the occurrence of single SSR, combined SSR and the most abundant motifs longer than 20 bp. We have found 91 dinucleotide, 171 trinucleotide and 224 tetranucleotide, 217 pentanucleotides and 106 hexanucleotides across 330 transcripts with a size superior to 150 bp. Sixty nine transcripts contained more than one microsatellite, and five transcripts contained 5 SSRs and one 6SSRs. Unexpectedly was the higher number of repeats containing tri, tetra penta and hexa units, being more abundant than for example dinucleotides SSRs. Although most of the sequences with SSR detected allowed primer design it will require further testing to determine their utility as markers. However our dataset increased the number of markers available for *R. decussatus* with the advantage of being linked to known genes, facilitating linkage map development or gene mapping.

**Table 1:** **Statistical analyses of EST-SSRs present in *Ruditapes decussatus *** **transcriptome**

**Sequences statistics**	**Number**	**%**	**Most predominant**
SSR containing:	330	1,0	
Containing > 1 SSR:	140	0,4	
With combined SSR	69	0,2	
Total	33247	100,0	
**SSRs and Distribution:**			
Dinucluotide	91	11,2	AT/AT (75)
Trincleptide	171	21,1	AAC/GTT (42)
Tetranucleotide	224	27,7	ACGT/ACGT (70)
Pentanucleotide	217	26,8	AACGTT/ACGTT (101)
Hexanucleotide	106	13,1	AACGTT/AACGTT (44)
Identified SSR (total)	809	100	AACGT/ACGTT (101)

### Rdecusdb, a *Ruditapes decussatus* database

*Rdecusdb* (http://morse-ccmar.ualg.pt/edge) is centered on contigs sequence and annotation. All contig sequences as well as different layers of results for data analysis will be available through Tripal [35]. Tripal is an open source and freely available collection of Drupal modules for management and visualization of data stored within a GMOD Chado database. Analysis results are indexed by Drupal’s full text searching mechanism, allowing the users to find data of interest. For each contig, a gene-like entry shows different data and bioinformatic analysis results, being identified with a description together with a sequence in fasta format along with blast hits and the reads that assembled that same contig. In addition, for each contig and whenever predicted, Gene Ontology is given for Biological Process (BP), Molecular Function (MF), and Cellular Component (CC). IT includes several analysis such as: the Analysis BLAST homology module, the Analysis InterPro module, the Analysis KEGG module; and the Analysis GO module for displaying trees and charts for GO mappings. Recently the sequences were update due to a new assembly. The assembly used in this article and the recent one can be download from the website.

### Comparison of gene expression in infected versus non infected clams

Clam gills were used because they are the main connection with the outside environment, together with the siphons which are one of the most affected tissues upon *Perkinsus* parasitism [36]. They participate as defense barriers sharing functions in the respiratory process and being also involved in the elimination of ROS molecules by endogenous antioxidant genes [37].

Data captured from transcripts fluorescence hybridization derived from four non infected and four infected clam gills was normalized and used to identify transcripts differentially expressed between the two conditions. Principal component analysis of conditions proved the good clusterization of samples (4 biological replicates) and consistence of the results between replicates, allowing the differences in steady-state mRNA levels between infected and non infected clams to be reliably measured.

All microarray data was deposited in the GEO database [38] under accession numbers GSE36276.

To perform microarray analysis a two unpaired class Significance Analysis of Microarray (SAM) test was carried out on normalized data. By imposing a False Discovery Rate (FDR) of 5% and Fold Change (FC) >1.5, a list of 949 probes, was obtained (see Additional file 3). From these, a total of 227 transcripts were up-regulated in infected clams versus non infected clams with a FC ranging from 1.5 to 102 while a total of 722 transcripts were down-regulated with a FC ranging from 1.6 to 76.

**Figure 4:**
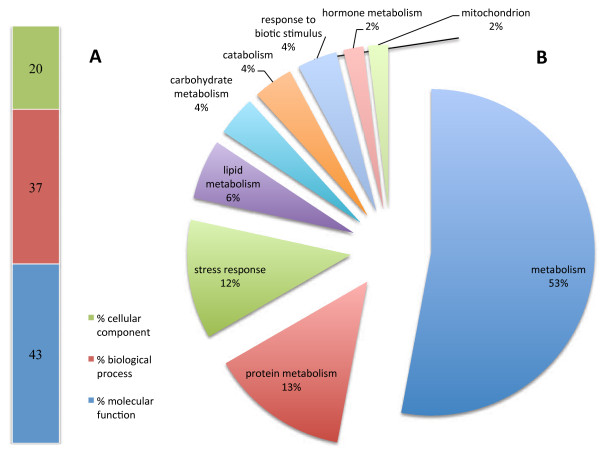
**Functional categories distribution of up regulated genes according to cellular component, biological process and molecular function (A) and immune class genes (**http://www.animalgenome.org/tools/catego/.goslim/immune_class**) (B).** Percentage of transcripts is reported for each functional category.

Genes were identified and categorized in terms of percentage using GO categories (cellular component, biological process and molecular functions) and specifically according to possible role in the immune response. For up regulated genes (Figure 4), more than half were found to be involved in general metabolism (53%) and in protein, lipid and carbohydrate metabolism (13%, 6% and 4% respectively) and in specific processes like stress response (12%) and response to biotic stimulus (4%). Expression of several genes associated with mitochondria represented two per cent of genes up regulated. Among the genes found to be down regulated, 46% were associated with general metabolism while only 1% was associated with lipid metabolism. For both categories, percentages were lower than those found for up regulated genes. Other gene clusters were also less represented when compared, such as those related with biotic stimulus (1.2%), stress response (7%) or even not present such as mitochondria related.

**Figure 5:**
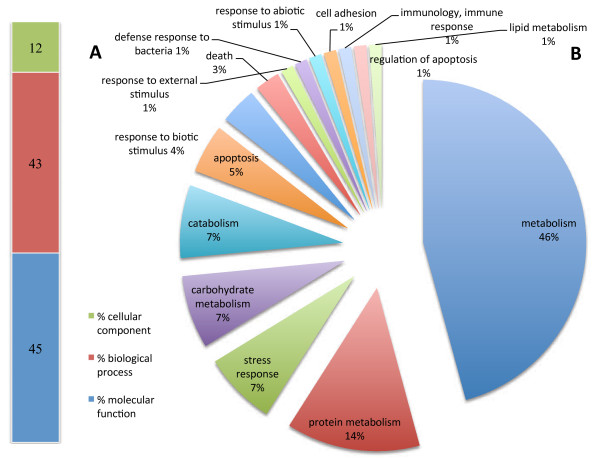
**Functional categories distribution of down regulated genes according to cellular component, biological process and molecular function (A) and immune class genes (**http://www.animalgenome.org/tools/catego/.goslim/immune_class**) (B).** Percentage of transcripts is reported for each functional category.

In contrast, the percentage of down regulated genes associated with apoptosis (1%), defense (1%), immune response (1%) and response to external/internal stimulus (2%) increased when compared with up regulated genes (Figure 5). Altogether, data suggests that metabolic and stress related genes are the most affected by Perkinsus parasitism, reflecting changes in growth and clam survival.

From all genes found to be differentially expressed upon Perkinsus infection (Additional file 3), only 67 of a total of 227 up regulated genes and 259 from a total 722 down-regulated genes were annotated successfully based on NCBI (National Centre for Biotechnology Information) amino acidic non redundant (nr) database. Some annotation was trivial and a second search using ncbi nr together with nt database was conducted, obtaining a more accurate annotation, returning 44 genes for up-regulated (Additional file 4), and 231 for down-regulated (Additional file 5). From these two lists we can identify lectins and a number of genes associated with immune/stress response. Up regulated genes include matrilin, a gene already associated with zebra mussel hemocytes host defense [39], methionine-r-sulfoxide reductase, a gene linked with antoxidant stress [40], exosome component 5, a set of genes capable of an immunomodulatory activity [41,42], different kinds of proteases (serine proteases), some already described as defenses against *Perkinsus* in different bivalves [43-49], acid phosphatases-like genes and dimethylarginine dimethylaminohydrolase, a gene associated with immune response in amphioxus [50].

Among the down regulated genes (see Additional file 5), we can point out several related to calcium binding such as calmodulin. This finding is in agreement with previous data. Indeed, one of the majors players during *Perkinsus* infection is hypoxia [51] and calmodulin is known to be down regulated during hypoxia events in mussels [52]. Calmodulin can be associated with almost all cellular processes, including apoptosis, metabolism, inflammation and the immune response. Some immune responses such NF-κB signaling pathway in pearl oyster are regulated by calmodulin binding proteins such as calcineurin [53] and calmodulin was shown to be an important molecular determinant response to *Perkinsus* infection [54,55].

The presence of lectins among the down-regulated genes constitutes an indication that *Perkinsus* parasitism can also negatively affect expression of some lectins as already shown for mussels [56] where multiples genes involved in immune defense are down regulated upon exposure to an infectious agent. Glutathione s-tranferases (GST) are also less expressed in this situation, a result contrary to our expectations since due to their role in cell detoxification and oxidative stress response, necessary for protecting the clam from the oxidative burst, we expected these levels to be up-regulated. However, and in agreement with our findings, some authors already demonstrated that in other mollusks, GST is up-regulated during the initial steps of infection but decreases when infection is established [57,58]. Other genes related with oxidative stress, such glutaredoxin a, aldo-keto reductase, hmgb-like protein, hephaestin, glucose dehydrogenase, peptide o-xylosyltransferase-like or agglutination (hemagglutinin amebocyte aggregation [59]) and anti-apoptosis related genes (e.g. achain structure of the ciap2 ring domain) were also down regulated, supporting the theory that after the initial infection period there is a relaxation of immune defenses.

**Figure 6:**
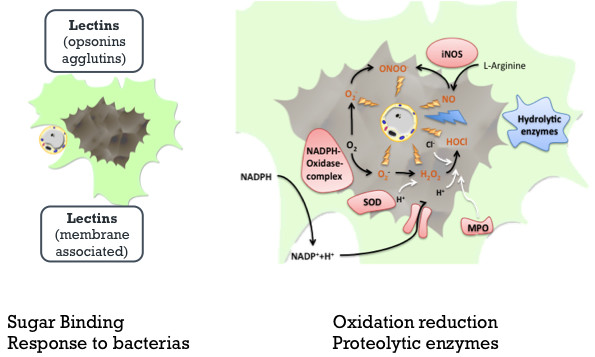
**Graphical representation of humoral and cellular responses of clams upon *Perkinsus *infection.** The two panels represent the initial recognition by lectins (left panel) and some response factors (right panel) associated with oxidative and proteolytic protection at intracellular level, like hydrolytic enzymes, Nitric oxide (NO); peroxynitrite (ONOO^-^); Anion superoxide O^2-^ Hypochloride (HOCl); inductible nitric oxide synthase (iNOS); superoxide dismutase (SOD); myeloperoxidase (MPO) (adapted from Soudant et al. 2008, 2009).

In order to obtain a more systematic functional interpretation of the set of differentially expressed genes, enrichment analyses using the Database for Annotation, Visualization, and Integrated Discovery (DAVID) was performed. Indeed, among the up-regulated cluster of ESTs, we found a high number of transcripts coding for immune response related genes as expected. The immune system of clams and bivalves in general is deprived of an adaptive system and fight pathogen aggression through an innate immune response [60] exerted by humoral factors and cell-mediated mechanisms (Figure 6). Humoral factors include lectins (agglutinins, opsonins), lysosomal enzymes (phosphatase acid, lysozyme and various hydrolytic enzymes), antimicrobial peptides and protease inhibitors, among others [61]. The constitutive or induced expression of such genes can potentially be directly linked to an effort to arrest *Perkinsus* infection as observed in other systems. It is interesting to observe that oxidative processes, hydrolase activity and nucleic acid binding are the main processes represented and most were already pointed out as having influence during resistance to microorganisms (Table 2). At the cellular level we could also highlight a number of genes linked to non-membrane bounded organelles and to intracellular non-membrane bounded organelles, suggesting that activity from these organelles (ribosome, cytoskeleton related) is necessary for the entrapment of microorganisms and for protein synthesis.

**Table 2:** **GO terms significantly over-represented, among genes differentially expressed, between *Perkinsus *** **infected and non infected clams**

**Category**	**Term**	**Count**	**%**	**P-value**	**Benjamini**
	Response to bacterium	5	3	4,1E-04	1,00E-01
**Biological process**	Translation	9	5,4	1,7E-03	2,00E-01
	Oxidation reduction*	11	6,6	3,3E-02	3,80E-01
	Hexose metabolic process	4	2,4	3,7E-02	6,70E-01
	Monosaccharide metabolic process	4	2,4	3,7E-02	6,30E-01
	Cytoskeleton organization	4	2,4	7,1E-02	6,80E-01
**Cellular compartment**	Nucleosome	3	1,8	2,2E-02	6,60E-01
	Protein-DNA complex	3	1,8	2,4E-02	4,60E-01
	Non-membrane-bounded organelle	10	6	4,1E-02	5,00E-01
	Intracellular non-membrane-bounded organelle	10	6	4,1E-02	5,00E-01
	Chromatin	3	1,8	7,8E-02	6,40E-01
**Molecular function**	Triose-phosphate somerase activity	2	1,2	1,8E-02	9,30E-01
	Hydrolase activity, acting on carbon-nitrogen (but not peptide) bonds, in linear amides	3	1,8	4,3E-02	9,60E-01
	Translation factor activity, nucleic acid binding	4	2,4	4,3E-02	8,70E-01
	Intromolecular oxidoreductase activity, interconverting aldoses and ketoses	2	1,2	8,8E-02	9,60E-01
	RNA binding	6	3,6	9,5E-02	9,40E-01
	Hydrolase activity, acting on carbon-nitrogen (but not peptide) bonds, in linear, amidenes	2	1,2	9,6E-02	9,10E-01
**KEGG pathway**	Glycolysis Gluconeogenesis	6	3,6	3,6E-04	2,10E-02
	Base excision repair	3	1,8	4,8E-02	7,60E-01
	Fructose and mannose metabolism	3	1,8	6,4E-02	7,30E-01
	Focal adhesion	6	3,6	7,4E-02	6,80E-01
	Nucleotide excision repair	3	1,8	7,6E-02	6,00E-01
	NOD-like receptor signaling pathway	3	1,8	9,7E-02	6,30E-01
**Sub category**	**Term**				
*Oxidation reduction	Aldo-keto reductase family member A1a (aldehyde reductase)				
	Dehydrogenase/reductase (SDR family) member 11a				
	Hypothetical LOC570613; sorbitol dehyfdrogenase				
	Methionine sulfoxide reductase 33				
	Similar to alcohol dehydrogenase S; alcohol dehydrogenase S				
	Sulfide quinone reductase-like (yeast)				
	Lbiquinol-cytochrome reductase hinge protein				
	Hydroxysteroid (17-beta) dehydrogenase 14				
	zgs: 56622; similar to CG6084CG6084-PA				
	Short chain dehydrogenase/reductase family 16C member Sa				
	Aldehyde dehydrogenase family, member A1				

### Importance of lectins on Host Parasite interaction

**Figure 7:**
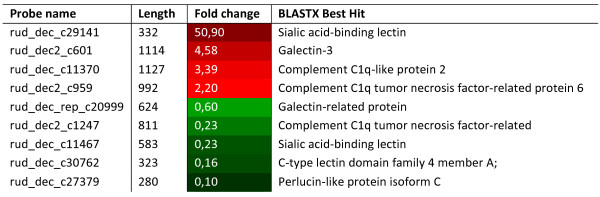
**Retrieval of keyword lectins in Rdecusdb with a significant fold change associated and respective size of the contig.** In red lectins up regulated and in green when is down regulated, between infected and non infected clams. Blastx best hit represents the closest homology when blasting the sequence against genbank database.

Due to the important role of lectins upon *Perkinsus* infection, this family of genes was chosen to be the subject of a particularly detailed characterization. Three different families of lectins were identified: C-type lectins, C1q-lectins and galectins. The members of these families were identified using conserved residues as patterns for search. A search into Rdecusdb revealed over than 90 sequences using lectin as keyword, but some genes were just classified as having a lectin domain. For classification purposes all the transcripts were organized in families and superfamilies and respective fold change annotated. Also the domain architecture and presence of signal peptide was deduced (see Additional file 6: figure S6). Nevertheless 9 of those genes (Figure 7) were differentially expressed when comparing infected with non infected clams, one of them being the identified gene with the highest fold change in the array (over 50 times over expressed).

**Figure 8:**
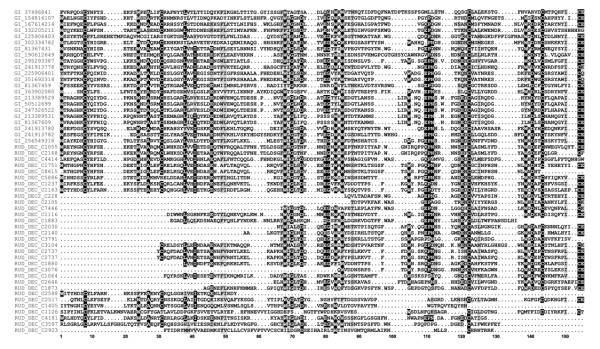
**Diversity of C-type lectins founded in *R. decussatus *sequences.** Alignment with other bivalve c-type lectins highlighted important residues. Cystein and Tryptophan residues are usually conserved (positions W1, C8, C31, W68, W83, W85, W105, C122, W139 and CC154) and together with other important residues such as A27, L38, G70, G82, D141 and E155. All positions shadowed are conserved at least 50%.

C-type lectins presented some conserved domains such WxD (position 139 and 141) [62] and CE (position 154) as shown in Figure 8. Cysteines are maintained due to their capability of forming disulphyde bonds [63], while WxD region is associated with calcium binding. Most of the lectins known to be linked to Perkinsus response are C-type lectins [10,64]. They have diverse carbohydrate specificities and present multiple structural domains. They can be classified in different groups including collectins, proteoglycan core proteins, selectins, endocytic receptors, and the mannose-macrophage receptor, some of them directly or indirectly involved in immune function [65].

Galectins, which constitute one family of lectins, are characterized by a conserved sequence motif in their carbohydrate recognition domain (CRD) and a specific affinity for b-galactosides. When compared with other bivalves galectins, *Ruditapes decussatus* galectins present some conserved signature residues F(D/N)XR(F/L/I), (N/K)X(V/I/L)XXN and WGXERXR [66]. Interestingly, despite the large evolutionary distance between invertebrate and vertebrates galectins, they still share more than 30% of homology. Host galectins are also known to be related to presence of parasite and in oysters it was previously shown that some galectins are induced following *Perkinsus marinus* infection [12].

**Figure 9:**
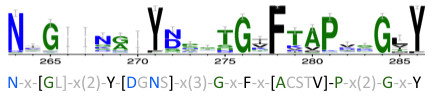
**Partial alignment of amino acid sequences from bivalves C1q family.** All sequences available were collected and most conserved region is presented as a logo, where highly conserved residues are shown as larger characters with error bars. A possible signature for identification of C1q sequences is underlined. Numbers indicate the amino acid position relative to the align consensus sequence.

C1q domain containing proteins are essential in the innate immune system of invertebrates, and can be the link between innate immune system and adaptive immunity as seen in lamprey, where mammalian homologous C1q was shown to be act as a lectin in lamprey [67]. The C1q has a globular domain [68] and is one of the most represented lectins. C1q can be involved in a variety of immune related processes such as activation of complement system or pathogen recognition and even mediating cell migration [69]. The *R. decussatus* Sialic binding lectins (C1q) namely rud_dec_c29141 were shown to be highly expressed in the presence of *Perkinsus olseni*. To improve c1q pattern recognition all sequences from bivalve C1q lectins deposited in Genbank were collected (August 2012) and conserved residues were analyzed, allowing us to identify a bivalve C1q signature (Figure 9).

## Conclusion

High throughput methodologies such as NGS and microarrays are changing our approach to biodiversity in a way that makes possible to understand changes in adaptation of a semi closed microcosm, like the one represented by a bivalve and its parasite (e.g. *Ruditapes* sp. and *Perkinsus* sp), at the transcriptomic level. This should allow us to acquire a broader understanding of all its molecular determinants, in particular those involved in host defenses and adaptation to parasite and thus contribute to unveil weak or advantageous host genetic characteristics in order to select more resilient clams. This approach will be of relevance to start a selection program based on specific genetic characteristics. In the case of bivalves, transcriptomic approaches can shed light on delicate issues for aquaculture such as the effect of pollutants, infections and human environmental interference. Clams can be very sensitive to these factors and the availability of new biomarkers can boost the use of clams as water quality biomonitors. This study is relevant for addressing the high mortalities associated to *Perkinsus sp.* in different mollusc species, and the consequent impact and losses at economic/social level.

Although clams can express specific cellular and humoral responses to *Perkinsus* infection, until now the invertebrate innate immune system might have been underestimated and over-simplified [63,70]. Yet, through the use of microarrays to assess the hosts’ immunological and physiological responses to *Perkinsus* infection, we could identify some factors related to the first line of defense against the parasite’s invasion. From this study we can conclude that a number of genes are altered by *Perkinsus* infection, in particular those belonging to important gene families such as lectins, which are part of the host defense system and capable of recognizing specific molecules through their carbohydrate recognition domains. Some of them were already characterized, such as the specific galectin that is used by the host to recognize *P. marinus* or others that are differentially expressed upon Perkinsus infection. In oyster, this specific galectin can be responsible for subverting the host's immune/feeding recognition mechanism, giving *Perkinsus marinus* a passively gain entry into the host hemocytes, the first line of defense [71]. These families of lectins were identified in *R. decussatus* and are now being subjected to a more detailed characterization. In *R. philippinarum* some lectins, known as Manila Clam Lectins (MCL) were also associated with *Perkinsus* infection and are able to bind to the surface of *Perkinsus* hypnospores, indicating that MCL plays a particular role in clam defense [12,62,72].

*P. marinus* and his most affected host, the oyster *C. virginica*, was already the subject of a similar microarray study [55] again emphasizing the importance of solving this problem that affects at the moment the bivalve production worldwide. In the case of *C. virginica* the response of the oyster was mainly at levels of antimicrobial and oxidative stress, consistent with the microarray results obtained for infected *R. decussatus*, and thus providing additional information on the molecular determinants involved in host interaction, by identifying the corresponding genes. Also Suppression Subtractive Hybridization was used to attest genes differentially expressed in *R. decussatus* during *Perkinsus olseni* infection [73] confirming that the major genes involved were related with immune and stress response.

Non annotated genes which expression was found to be substantially altered during *Perkinsus* infection are also being the focus of specific analyses and in a near future we expect that they will be identified and their role during that process documented. Nevertheless, the most important aspect of this study is its contribution to increase the number of ESTs available for bivalves genetics studies. It is also expected to provide tools to infer some facts like how pollution and human related activities can influence clams transcriptome or the effect of some biological events such as metamorphosis or reproduction.

Although we believe to have a good representation of *Ruditapes decussatus* transcriptome, and despite the use of normalized libraries of adult and larvae stages, the representation of the clam transcriptome is incomplete and some of the contigs are not fully represented, often missing its 5’ extremity, a problem associated with cDNA library construction. The continuous technological advances and NGS cost drop should provide, in a near future, full coverage of relevant transcriptomes for this and other organisms, allowing the identification and use of more biomarkers or defense related genes to characterize different populations of bivalves of a specific area. Furthermore they should allow us to also identify the specific adaptations of each species to adverse and/or favorable conditions. But already at present, with the availability of two *Ruditapes* species transcriptomes (this study and Milan and collaborators [24]), we can start to identify the genetic differences behind their susceptibility to different pathogen organisms and point out resistance factors.

## Methods

### Sampling, cDNA library construction and sequencing

Samples of *R. decussatus* were collected in Faro, in the Ria Formosa lagoon system which spreads along the mid region of the southern Portugal coast. Total RNA was extracted using the acid guanidinium thiocyanate-phenol-chloroform method [74]. Two libraries were constructed, one using a mixture of all adult tissues from 20 individuals and a second using gonadal tissues from both sex with a ratio of 1 male to 4 females (gonadal phase IV) and juveniles clams with sizes ranging from 2 to 4mm total length. The cDNA libraries were constructed using the SMART kit from BD Biosciences Clontech and equal amounts of RNA and then normalized using the duplex-specific nuclease (DSN) method [75].

Sequencing was performed at the Max Planck Institute using 454 GS FLX instrument with Titanium series chemistry following manufacturer protocol. Pyroluminescence intensity was converted to sequence data using Newbler suite. 454 reads were post processing using sff_extract (0.2.8) and trimmed using clean_reads (0.2). Final reads quality was assessed using prinseq-lite (0.14.4).

### Transcriptome assembly

A hybrid assembly using ESTs collected from Genbank and all 454 reads was performed to improve the assembly. For the latter the quality score files were taken into consideration. The purpose of a hybrid assembly is to explore the advantages of the two technologies, the numerous reads of 454 and the quality and length size of Sanger reads. MIRA3 performed the assemblies in two runs [76], where all contigs obtained with the first run of hybrid assembly were used for a second run to eliminate contig redundancy.

### Transcripts annotation

Little information, specifically on gene annotation, is available in public databases for mollusks species, with the exception of recent deposition of sequences from Pacific oyster [23,77], blue, Mediterranean [56] and deep sea vents mussel [78] and manila clam [24,79]. Although those species are not annotated in a satisfactory way, they can still provide some extra information. Blast searches were conducted against NCBI (National Centre for Biotechnology Information) amino acidic non redundant (nr) database (release of March 2012), using Blastx option. Alignments with an E-value of at most 1.0 e^-10^ were considered significant, and up to 10 hits per contig were taken into account.

Unfortunately, like any non-model organism, the annotation of the clam transcriptome can be a challenge and the annotation project was conducted using two other different strategies by i) blasting against ensemble protein databases of different species including *Danio rerio, Gasterosteus aculeatus, Oryzias latipes*, *Takifugu rubripes*, *Tetraodon nigroviridis*, *Homo sapiens, Drosophila melanogaster* using a cutoff value of <1.0 e^-5^], and ii) blastn search (cut off e-value of <1.0 e^-5^) against *Lottia gigantea* v1.0 database [80], *Crassostrea gigas* transcripts databases [23,81] and *Argopecten irradians* EST database [82].

For de novo annotation of *R. decussatus* contigs, we used Blast2go tool, which encompasses all the tools for functional annotation of (novel) sequences and the analysis of annotation data [83,84]. The Gene Ontology (GO) terms associations for BP, MF and CC were performed using Blastx algorithm against the NCBI amino acid nr database implemented in Blast2GO software. To categorize the GO terms into different GO categories, a web-based tool, CateGOrizer [85], was employed.

### EST-SSR search

Misa software was used to screen simple SSRs and msatcommander for complex forms (combinations of different SSRs of coexistence of two or more SSRs). In either cases search was performed to obtain SSRs longer than 20 bp and at with the following tuning: dinucleotide repeat ≥ 20 bases; trinucleotide repeat ≥ 21 bases; tetranucleotide repeat ≥ 20 bases; pentanucleotide repeat ≥ 20 base; hexanucleotide repeat (HNP) (and more) ≥ 24 bases.

### DNA microarray design

**Figure 10:**
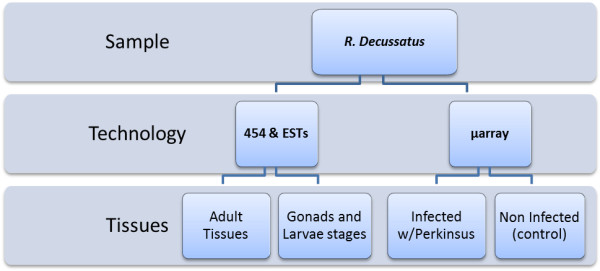
Overview of tissues origin and sequencing technologies used in this report.

Agilent technology of oligo-DNA microarray was chosen to design a specific microarray based on sequenced transcriptome (Figure 10), containing two probes with both orientations considering all annotated transcripts and unknown transcripts with Phred quality>30 and length>400 bp. This 60mer oligo-probes design, in a 4 × 44K format, was assisted using the Agilent eArray interface [86].

### Biological handling, RNA extraction, labeling and hybridization

Fifty grooved carpet shell clams with a size of 25-28 mm were collected from the wild in the Ria Formosa, Portugal and rested in proper aquariums for 4 days before sacrifice in order to reduce stress. Gills were dissected and immediately homogenize in Tri-reagent (Ambion, Austin. USA) and simultaneous a small portion of the gills and the rest of the tissues was incubated in Ray's fluid thioglycollate medium assay, following previously established protocol [4], to determine the level of Perkinsus.

Two groups were selected, one of four clams not infected (No hypnospores present) and another with four clams heavy infected with hypnospores presented in all tissues (Mackin scale 5). RNA was extracted individually from these two groups using Tri-reagent (Ambion, Austin. USA), following manufacturer’s instructions and later purified and treated with Dnase I using the RNeasy Mini Kit (Qiagen, Hilden, Germany), following the manufacturer’s instructions for RNA cleanup. RNA concentration was determined using a NanoDrop® ND-1000 spectrophotometer, (NanoDrop Technologies, Wilmington, USA) and integrity and quality were finally evaluated on an Agilent 2100 Bioanalyzer (Agilent Technologies, Palo Alto, CA).

Labeling was done using 200 ng of total RNA linearly amplified and labeled with Cy3-dCTP (Agilent One-Color Microarray-Based Gene Expression Analysis). For control, a mixture of 10 different viral poly-adenylated RNAs (Agilent Spike-In Mix) was added to each RNA sample before amplification and labeling. Labeled cRNA was purified with Qiagen RNeasy Mini Kit, and sample concentration and specific activity (pmol Cy3/μg cRNA) were determined using a NanoDrop spectrophotometer. A total of 1,650 ng of labeled cRNA was prepared for fragmentation adding 11 μl 10X Blocking Agent and 2.2 μl of 25X Fragmentation Buffer, heated at 60°C for 30 min, and finally diluted by adding 55 μl of 2X GE Hybridization buffer. A volume of 100 μl of hybridization solution was then dispensed in the gasket slide and assembled to the microarray slide (each slide containing four arrays). Slides were incubated in the oven overnight at 65°C and then washed according to manufacturer’s protocol.

### Microarray scanning and data processing

Scanning was performed twice at two different sensitivity levels (XDR Hi 100% and XDR Lo 10%) at 5 μm resolution using an Agilent G2565BA DNA microarray scanner. The two images were analyzed together and data were extracted and background subtracted using Agilent Feature Extraction (FE) Software version 9.5.1. After quality measures, all control features (positive, negative, etc.), except for Spike-in (Spike-in Viral RNAs), were excluded from subsequent analyses. Normalization procedures were performed using R statistical software using Spike-in control intensities to normalize each dataset. Significance Analysis of Microarray (SAM) [87] was used to identify differentially expressed genes between healthy clams and those infected with *Perkinsus*.

### Functional enrichment of differentially expressed genes

Gene functional annotation based on gene enrichment was performed using the Database for Annotation, Visualization and Integrated Discovery (DAVID) v6.7 [88,89]. DAVID is capable of recognizing functional annotation data from limited species, mainly human, mouse and zebrafish. So, in order to use clam data, it was necessary to convert clams genes into the equivalent orthologs of zebrafish Gene IDs or entrez entries, by blasting clam nucleotide sequences against zebrafish protein counterparts. This was done by downloading blasted annotated protein sequences and performing in-house blast routines. Then gene ontology search was performed in DAVID using two lists, one with all identified genes as background and another with the differentially expressed up and down regulated genes using the same predefined settings.

### Multiple sequence alignments

Available c-type lectins and c1q sequences were retrieved from bivalve species present in Genbank, including *Mytilus sp., Haliotis sp.*, *Chlamys farreri*, *Mercenaria,* and *Ruditapes philippinarum* and aminoacid protein sequences aligned using T-Coffee server [90], applying default settings. Alignments were subject to a posterior manual adjustment. C1q signature was determined using PRATT [91] server and sequence logos were then created from multiple alignments using WebLogo [92].

### Architecture domain analysis

Domain analyses of lectins present in *R*. *decussatus* was performed using Superfamily at http://supfam.cs.bris.ac.uk/SUPERFAMILY[93] using nucleotide sequences of putative lectins discovered in our database. Lectins were classified according to domain architecture. Signal peptide was predicted using SignalIp 4.0 server [94].

### Quantitative real-time PCR analysis

Quantitative real-time reverse-transcription polymerase chain reaction (qRT-PCR) was performed to validate and assess the microarray data. Primers were designed for 12 differentially expressed genes identified by microarray analysis (Table 3), randomly chosen. Real-time qPCR was performed in a StepOnePlus apparatus (Applied Biosystems) using gene specific primer sets to quantify expression of selected genes. Each reaction was prepared by adding 2 μl of a 1:10 cDNA dilution to reaction mix containing 0.2 μM of each primer and 10 μl of SsoFast™ EvaGreen® (Bio-Rad), in a final volume of 20 μl. The qPCR program contained an initial cycle of 10 min at 95°C followed by 45 cycles comprising an initial denaturation step at 95°C for 20 sec then annealing and extension at 68°C for 15 sec. The fluorescence was measured at the end of each extension cycle in the FAM-490 channel. Relative levels of clam gene expression were determined by 2^–ΔΔCt^[95] comparing non infected versus infected conditions and normalized with a previous described housekeeping gene for *R. decussatus,* the L28 ribosomal gene [96]. The neutral behaviour of this gene was also confirmed in the microarray data. PCR efficiency was determined for each pair of primers by using at least 4 different dilutions of the template cDNA and all the primers pair showed efficiency between 89 and 96%. All experiments were performed at least twice, and in a minimum of triplicate wells. Microarray fold change was compared with relative gene expression of Realtime qPCR in order to validate microarray results.

**Table 3:** Real time Primers used for microarray validation

**Putative gene annotation**	**Sequence identifier**	**Primer sequence 5’->3’**	**Amplicon lenght (bp)**	**PCR efficiency**
N.D	rud_dec_c1521	F-GCACTTGTTGGTGGTCCTTATTGCTGG	183	92%
		R-TGATTTGGTTAGTCAACTTCGCCG		
Sialic acid-binding lectin	rud_dec_c29141	F-CGTGGGCAGAACCTTTCAGTATGAG	98	94%
		R-CACTTCACCCACACCCGTTGTCCTT		
Pyridine nucleotide-disulphide	rud_dec_c15726	F-CCTGGGGAGCCGTCATCATTAGC	149	91%
		R-TACCTCCCCTTCTCTTCCCAAAACAA		
Tetraspanin,	rud_dec_c39872	F-TGGGTTCGGTAGTTTTGTCCTTCTAGTC	153	95%
		R-AAAACTGCTGCTGTAATACCACCCGAG		
Epididymal secretory protein 1	rud_dec_c2016	F-ACCTGTTCCATTTCCCGTTCCCT	149	95%
		R-GGACTTCCCACTTTACAAGCAGCCG		
Methionine sulfoxide reductase	rud_dec2_c944	F-AGATACCAAGTTCAACTCCCACTCGG	261	89%
		R- CATCAGCCAGTGTTACGCTTTC		
X- box binding protein 1	rud_dec2_c33	F-CAAGCAATCGCAAATCGCCAACA	225	92%
		R-GTGGGAGACACTTTAAGTTGACCAG		
Fructose-bisphosphatase	rud_dec_c1253	F-AAGCAGCGGAACAGGCTAAAGAG	199	90%
		R-CTGCCAGTGCTCTAAATGCCTTGTT		
Inhibitor of apoptosis protein	rud_dec_c1093	F-ATTGCCTGTGGTCACATGGTTA	110	96%
		R-AGACAGCCATAAGAGCACGGACA		
Glucose dehydrogenase	rud_dec_c2753	F-TGGGAATGTTTCGTTCGTCACCT	146	93%
		R-GAGGCATTCAACAACTCGAACC		
ND	rud_dec_c27604	F-GAATACTGCTTGTTGCTTTCGGTGT	102	95%
		R-TGCCTCTCACTTCGTCTGTGTCGGA		
Calmodulin	rud_dec_c873	F-TGAAGTTGTATGCTGACGGAAATGGA	111	95%
		R-TTGGAATACTTCAAGTAACCCCTCTTCACTA		

## Competing interests

The authors declare that they have no competing interests.

## Authors’ contributions

RL, LB, MM, LC, TP and CS conceived and designed the project. AC and SB have assembled 454 reads. RR produced the EST sequences. AJ conceived and constructed the database. MM carried out probe design and editing, and RL and MM performed microarray experiments. RL and MM executed all statistical analyses. RL performed functional annotation analyses. RL wrote the manuscript. All listed authors edited the manuscript. All authors read and approved the manuscript.

## Supplementary Material

Additional file 1: Table S1 Summary of Blastx (E-value < 10e-3) and Blastn (E-value < 10e-5) similarity searches on several protein and nucleotide databases for *R.decussatus* transcripts annotation. Click here for file

Additional file 2: Figure S2 Functional categories distribution of *R. decussatus* trancriptome, according to cellular component, biological process and molecular function (A) and using map2GO classification clustering (http://www.geneontology.org/external2go/egad2go). Percentage of transcripts is reported for each functional category. Click here for file

Additional file 3: Table S3 List of significant up and down regulated sequences identified by SAM analysis (Fold change>1.5; FDR=5%) by comparing controls and infected *R. decussatus*. In Green transcripts down regulated and in red transcripts upregulated. Click here for file

Additional file 4: Table S4 GO assignment, sequence description and first e-value of blasted sequences (Genbank nr/nt database) with a manual curated annotation (e-value < 1e-10) for up regulated genes. Click here for file

Additional file 5: Table S5 GO assignment, sequence description and first e-value of blasted sequences (Genbank nr/nt database) with a manual curated annotation (e-value < 1e-10) for down regulated genes. Click here for file

Additional file 6: Table S6 Retrieval of keyword lectins in Rdecusdb and classification of lectins according to Superfamily prediction. Sequences are distributed according to sequence name, superfamily and support e-value, family and respective support e-value and domain architecture. Colors represent classification, being yellow for c-type lectins, blue for C1q (TNF-like), green for galactose binding lectins, pink for Fibrogen C-terminal domain like, red for galectin and light cyan for Scavenger receptor cysteine rich domain. Black triangles denote the presence of a signal peptide and FC represents Fold change in the actual study. Click here for file
