# Supplementary figures and images for: mRNA-Seq and microarray development for the Grooved carpet shell clam, Ruditapes decussatus: a functional approach to unravel host -parasite interaction
Source: BMC Genomics. 2013 Oct 29;14:741. doi: 10.1186/1471-2164-14-741 (PMC4007648; doi:10.1186/1471-2164-14-741)

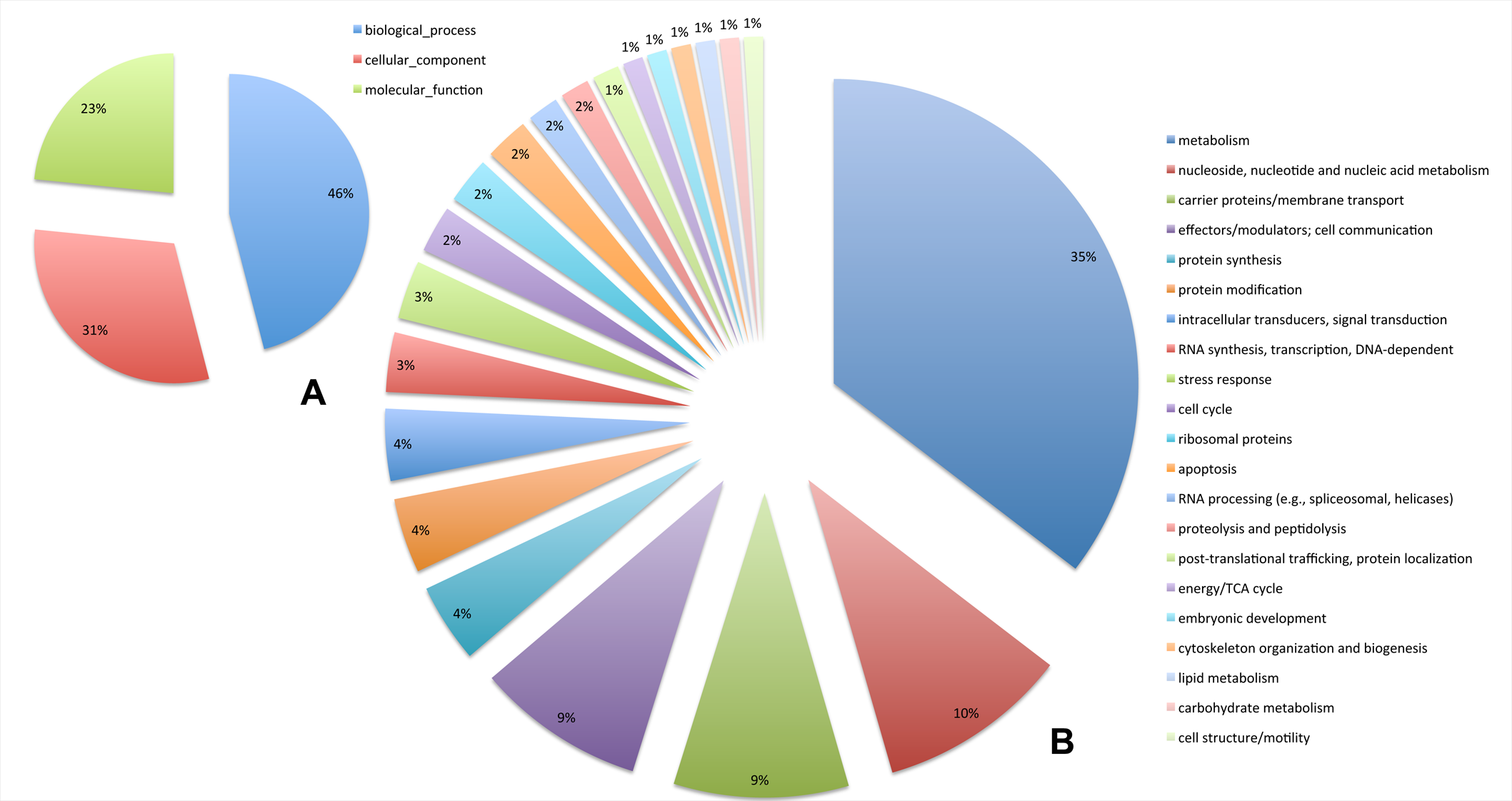

Supplement: Additional file 2: Figure S2 — Functional categories distribution of R. decussatus trancriptome, according to cellular component, biological process and molecular function (A) and using map2GO classification clustering (http://www.geneontology.org/external2go/egad2go). Percentage of transcripts is reported for each functional category. [file 1471-2164-14-741-S2.tiff]
